# Chemical Transfers Occurring Through *Oenococcus oeni* Biofilm in Different Enological Conditions

**DOI:** 10.3389/fnut.2019.00095

**Published:** 2019-06-25

**Authors:** Christian Coelho, Régis D. Gougeon, Luc Perepelkine, Hervé Alexandre, Jean Guzzo, Stéphanie Weidmann

**Affiliations:** ^1^UMR A 02.102 PAM Laboratoire PCAV AgroSup Dijon, Université de Bourgogne, Institut Universitaire de la Vigne et du Vin Jules Guyot, Dijon, France; ^2^SAAT Sayens, Maison Régionale de l'Innovation, Dijon, France; ^3^UMR A 02.102 PAM Laboratoire VAlMiS AgroSup Dijon, Université de Bourgogne, Institut Universitaire de la Vigne et du Vin Jules Guyot, Dijon, France

**Keywords:** malolactic fermentation, chemical transfers, wood, *O. oeni*, biofilm, planktonic, optical indices

## Abstract

Chardonnay wine malolactic fermentations were carried out to evaluate the chemical transfers occurring at the wood/wine interface in the presence of two different bacterial lifestyles. To do this, *Oenococcus oeni* was inoculated into must and wine in its planktonic and biofilm lifestyles, whether adhering or not to oak chips, leading to three distinct enological conditions: (i) post-alcoholic fermentation inoculation in wine in the absence of oak chips, (ii) post-alcoholic fermentation inoculation in wine in the presence of oak chips, and (iii) co-inoculation of both *Saccharomyces cerevisiae* and *O. oeni* directly in Chardonnay musts in the presence of oak chips. Classical microbiological and physico-chemical parameters analyzed during the fermentation processes confirmed that alcoholic fermentation was completed identically regardless of the enological conditions, and that once *O. oeni* had acquired a biofilm lifestyle in the presence or absence of oak, malolactic fermentation occurred faster and with better reproducibility compared to planktonic lifestyles. Analyses of volatile components (higher alcohols and wood aromas) and non-volatile components (Chardonnay grape polyphenols) carried out in the resulting wines revealed chemical differences, particularly when bacterial biofilms were present at the wood interface. This study revealed the non-specific trapping activity of biofilm networks in the presence of wood and grape compounds regardless of the enological conditions. Changes of concentrations in higher alcohols reflected the fermentation bioactivity of bacterial biofilms on wood surfaces. These chemical transfers were statistically validated by an untargeted approach using Excitation Emission Matrices of Fluorescence combined with multivariate analysis to discriminate innovative enological practices during winemaking and to provide winemakers with an optical tool for validating the biological and chemical differentiations occurring in wine that result from their decisions.

## Introduction

Microbial biofilms are three dimensional complex and dynamic systems composed of one or several species of microorganisms attached to a surface and surrounded by a self-produced extracellular matrix. They regulate biogeochemical transformations in environmental substrates and are involved in biotechnological applications in medical and industrial fields (1, 2).

In the agro-food industry Clean-in-Place procedures are used to maintain satisfactory sanitary conditions. However, despite these procedures, undesirable spoilage microorganisms may persist, especially when they have developed biofilm structures (3–5). This persistence is mainly due to the particular resistance properties of the bacteria developed under the biofilm phenotype against environmental stresses. Over the last decade, attention has focused on these properties concerning functional bacteria. Indeed, modern biotechnological approaches take advantage of biofilms to promote health benefits, improve food quality, and offer new methods of technological control over industrial processes (6–8). Microbial biofilms are of primary concern in the food industry since they can develop on any kind of surface in contact with food products (rubber, stainless steel, polyvinylchloride, polyurethane, and wood) (9, 10).

Among these beneficial microbial biofilms, lactic acid bacteria are of interest as they enhance the food quality of products and resist environmental stresses (6, 8, 11–14). In fermented beverages, particularly in red and white wines, malolactic fermentation (MLF) is a means of reducing wine acidity and improving aromatic equilibrium, and generally occurs just after alcoholic fermentation. This second fermentation is performed by lactic acid bacteria and especially *Oenococcus oeni*. Indeed, several species of lactic acid bacteria can perform MLF. However, *O. oeni* resists the harsh conditions of wine well (15) due to its acidophilic properties and tolerance to high concentrations of ethanol (16, 17), making it the species most often used as a commercial starter (18). Indeed, strains of *O. oeni* are selected for their capacity to consume malic acid and carry out certain enzymatic activities (19). They can be subjected to a pre-acclimation process to resist in particular ethanol levels, acidity, SO_2_, and certain stages of the preparation process, such as lyophilization. However, despite the efficiency of *O. oeni*, spontaneous MLF is difficult to predict and sometimes fails to occur due to the elevated mortality rate of the bacterial ferment exposed to the severe physicochemical conditions in wine. To counteract the deleterious effect of this environment, winemakers increasingly need to use commercial starters. Recently, an alternative method using *O. oeni* cells organized in biofilms on oak chips has shown unsuspected functionalities in wine, with increased tolerance to wine stresses, malolactic activities, and the modification of wood volatile composition in the resulting wines (6).

Knowledge of the chemical composition of microbial biofilms enables imagining alternative biotechnological practices involved in food elaboration and/or cleaning operations. It has been shown that small chemical units like lactones and larger biomolecules like extracellular RNAs released by microorganisms for cellular communication, induce the formation of biofilms (20–22). However, in environmental and food systems, natural phenolic and tannin compounds strongly inhibit biofilm-related genes and act as antibiofilm agents (23). Small peptides like nisin produced by *Lactococcus lactis* also present antimicrobial activities (21). Other active compounds, such as lectin-like proteins enable cell aggregations between the outer membranes of other microbial species like *Saccharomyces cerevisiae* (24). Secreted biomacromolecules, such as exopolysaccharides are regulated genetically by sessile cells to produce a well-ordered protective barrier organized in micro channels, favoring cell community persistence by exposing binding sites to other cells (25) and facilitating liquid transport for nutrients and wastes (26). The diffusion of chemical compounds through the biofilm matrix has also been investigated, with the observation of the presence of positively and negatively charged sites in biofilm exopolymers, leading to ionic adsorption through electrostatic interactions and ion-exchange mechanisms (27). Molecular transport across the biofilm has also been described, using diffusion theory and suggesting the use of relative effective diffusion coefficients to discriminate solute chemical mobility. In this theory light gases diffuse more than twice as fast as charged species or organic solutes, which are poorly differentiated in terms of their diffusing properties (28).

In this study, the technology of *O. oeni* biofilms developed on oak chips was extended to analyze different steps of wine elaboration processes and to focus not only on the transfers of wood volatile compound but also on grape and fermentative compounds to obtain a more exhaustive view of the chemical transfers occurring though bacterial biofilms and capable of affecting wine composition and final quality. The objectives of the study were therefore to evaluate the impact of bacterial biofilms on the chemical composition of wine and propose a fluorescence-based real-time analytical tool to diagnose the chemical modifications caused by biofilm during wine elaboration.

## Materials and Methods

### Wine Fermentation Conditions

Chardonnay grapes were hand harvested at the end of September 2016 at the “Domaine viticole de l'université de Bourgogne.” Whole grapes were pneumatically pressed prior to settling over night. Must turbidity was 80 NTU. The inoculation of *Saccharomyces cerevisiae* yeast at a dose of 20 g·hL^−1^ (Fermol® Chardonnay, AEB) was applied for alcoholic fermentation (AF). Three different conditions were maintained in 1 hL stainless steel wine containers to evaluate the role of *O. oeni* biofilm lifestyle (BF) in the completion of malolactic fermentation (MLF) compared to that of planktonic lifestyle (PAD). Two of these conditions were imposed immediately after alcoholic fermentation.

An industrial strain of *O. oeni* (Biolaffort) was cultured for 6 days in planktonic (floating) and sessile (biofilm) lifestyles. The growth medium was used according to the manufacturer's instructions (Biolaffort). Before inoculation in wine, the planktonic culture was adapted to the wine using the “pied de cuve” method (29), whereas the biofilm culture was inoculated directly.

Three winemaking conditions were tested and are summarized in Table 1.

**Table 1:** Chardonnay wine fermentation conditions imposed in this study by changing *O. oeni* lifestyle at the moment of addition and the presence/absence of oak during wine elaboration.

	**PAD 1**	**BF 1**	**PAD 2**	**BF 2**	**PAD3**	**BF 3**
*O. oeni* addition (10^6^ CFU·mL^−1^)	Post-AF	Post-AF	Post-AF	Post-AF	Pre-AF	Pre-AF
Oak (5 g·L^−1^)	Absent	Absent	Present	Present	Present	Present

*PAD and BF represent the bacterial physiological lifestyles acquired by O. oeni, in adapted planktonic and biofilm form in our three oenological conditions*.

In Condition 1 (BF1/PAD1), 6 days sessile *O. oeni* isolated from the stainless steel square support were prepared as described by Bastard et al. (6), but by increasing the volume of preparation using a fermenter dedicated to biofilm culture. Briefly, surface areas of 25 × 25 mm stainless steel chips were immersed in Biolaffort culture medium inoculated at 2 × 10^7^ CFU·mL^−1^. After 3 and 6 days, the cells developed in the biofilm on the chips were recovered by sonication (2 min at 40 kHz). The cells were either numbered [according to Bastard et al. (6)] or lyophilized (−80°C 3 h, 1,650 mbar) for further introduction in the wine after alcoholic fermentation (post-AF) at a concentration of 10^6^ CFU·mL^−1^. In parallel, a planktonic culture grown for 6 days in the Biolaffort medium was inoculated at 10^6^ CFU·mL^−1^ after AF.

In Condition 2 (BF2/PAD2), *O. oeni* biofilms developed as above for 6 days on 25 × 25 mm untoasted oak chips (6) were introduced directly in the wine at a concentration of 10^6^ CFU·mL^−1^ for bacteria and 5 g·L^−1^ for oak chips, and were compared to the planktonic form added to the wine in the presence of inert untoasted wood chips at the same bacteria concentrations of 10^6^ CFU·mL^−1^ and an oak chip concentration of 5 g·L^−1^. The number of oaked biofilm and planktonic cells was determined precisely by following the counting procedure reported previously (6).

For Condition 3 (BF3/PAD3) the same protocol as described in Condition 2 was applied by co-inoculating biofilm or planktonic *O. oeni* at 10^6^ CFU·mL^−1^ in the must containing oak chips at 5 g·L^−1^, together with *Saccharomyces cerevisiae* yeast at 20 g·hL^−1^ (Fermol® Chardonnay, AEB). Table 1 gives the three BF/PAD conditions used in this study.

### Routine Analysis During Fermentation

Classical enological parameters of the initial must and the wine just after alcoholic fermentation were monitored by FT-IR spectroscopy (OenoFoss™ analyzer). Initial concentrations are indicated in Table 2 for the must and wine used for this experiment. These values evolved during fermentation and are shown in Table S.I.1.

**Table 2:** Enological parameters of must used for Condition 3 and wine at the end of alcoholic fermentation used for Conditions 1 and 2 for the BF/PAD experiment.

	**Must (pre-AF)**	**Wine (post-AF)**
Ethanol (% (v/v))	0	12.73
Total acidity (g·L^−1^ Tartaric acid)	7.2	7.62
Malic acid (g·L^−1^)	3.7	1.9
Glucose (g·L^−1^)	205	0
pH	3.2	3.1
Volatile acidity (g·L^−1^ Acetic acid)	0	0.33

Viable planktonic and sessile bacteria were enumerated across wine fermentations by spread plating after serial dilution on modified MRS agar (MRS Broth (Laboratorios Conda Spain), 50 g·L^−1^; fructose 10 g·L^−1^; L-malic acid 4 g·L^−1^; agar 25 g·L^−1^; pH adjusted to 4.8). The plates were incubated at 30°C for 5 days under anaerobic conditions.

We routinely monitored wine fermentation by acquiring the Excitation-Emission Matrix of Fluorescence (EEMF) of musts and fresh fermented wines using 3D-fluorescence, with a standardized method described previously (30). All the samples were diluted 40 times with ultrapure water, placed in 1 cm path-length quartz cuvettes and analyzed with an Aqualog unit (Horiba Jobin Yvon, Inc.), by setting the excitation wavelengths from 225 to 600 nm (3 nm interval) and the emission wavelengths from 200 to 600 nm (3.22 nm). All the EEMF were corrected daily for Rayleigh scattering and inner filtering effects and finally normalized to 1 ppm quinine sulfate standard. A PARAFAC model was built using the Matlab drEEM box, published previously (31), from which a 4-PARAFAC-component model (C1, C2, C3, and C4) was derived, validated by a core consistency of 90% and split-half validated on four splits.

### Chemical Analysis of Finished Wines

#### Fermentative Component

The fermentative component of wines was estimated by quantifying the major higher alcohols present in the finished wines (propan-1-ol, isopropanol, 2-methylpropanol, butan-1-ol, butan-2-ol, 2-methylbutanol, 3-methylbutanol). Individual alcohols were determined by GC-FID (Varian, Inc.), using a direct 1/1,000 split injection of 1 μL of the sample into a CP-WAX 50 × 32 capillary column in the presence of octan-2-ol as internal standard. A temperature programme from 25 to 240°C was used in the column oven and maintained at a constant temperature of 25°C for 5 min, followed by an increase of 5°C·min^−1^ from 5 to 12 min and 15°C·min^−1^ from 12 to 24 min. The injector temperature was fixed at 240°C and the temperature of the flame ionization detector was set at 250°C. The methodology had been described previously by the Office International de la Vigne et du Vin (32). All the higher alcohols were integrated based on their area in the chromatogram and transformed into concentrations expressed in mg·L^−1^, before being summed to obtain the fermentative component.

#### Grape Component

Grape polyphenols were separated and quantified on an Acquity Waters UPLC-DAD-fluorometer. The column was a BEH C18, 1.7 μm, 2.1 × 150 mm, protected by a guard column packed with the same material. The column temperature was kept constant at 30°C, and the samples were maintained at 8°C. Elution started isocratically from 100% A (ultrapure water, 0.1% formic acid) from 0 to 6 min, and then increasing linearly over 56 min to 100% B (methanol, 0.1% formic acid), where it was maintained until 60 min, with a 0.3 mL·min^−1^ flow rate. The injection volume was 5 μL. Wine polyphenol concentrations were determined following calibration performed on a polyphenol mixture.

The grape polyphenolic component was evaluated by analysing the phenolic acids, the cinnamic acids, the flavan-3-ols and the Grape Reaction Product (GRP). Caffeic, coumaric, chlorogenic, ferulic, caftaric, and coutaric acids belonging to the cinnamic acid family were summed to obtain an equivalent concentration of cinnamic acids expressed in mg·L^−1^. Gallic, protocatechuic, hydroxybenzoic, gentisic, and salicylic acids belonging to the phenolic acid family were summed to obtain an equivalent concentration of phenolic acids expressed in mg·L^−1^. Catechin, (2)-epicatechin, dimer B1 and B2 belonging to the flavan-(3)-ol family were summed to obtain an equivalent concentration of flavan-3-ols expressed in mg·L^−1^. The concentrations of the phenolic acids, cinnamic acids, flavan-3-ols and GRP families were summed in order to obtain the grape component of white wines, expressed in mg·L^−1^.

#### The Wood Component

Five mL of 1 month aged wine was placed in a 20 mL sealed headspace vial (Supelco, Bellefonte, USA). The methodology was adapted from Bastard et al. (6). Headspace vials were then placed in the agitator/incubator of an automatic headspace sampler (GERSTEL MPS 2, Gerstel Inc., Mülheim an der Ruhr, Germany) and incubated at 70°C for 10 min (incubation time) to promote volatile compounds in the headspace. Extractions were performed by immersing a DVB–CAR–PDMS fiber in the headspace for 60 min (extraction time). After each extraction, the extracted compounds were desorbed at 260°C for 7 min in the injection port of an HP 6890GC equipped with an MSD 5973 mass detector (Agilent Technologies, Palo Alto, CA). The calibration solutions were processed in the same way using 5 mL of the wine matrix mixed with target compounds. Volatile compounds (eugenol, guaiacol, furfural, vanillin, cis-, and trans-whisky lactone) were purchased from Sigma-Aldrich and used as received. We used 3,4-dimethylphenol as an internal standard at 10 mg·L^−1^ in each sample and checked that there were no competition effects between aromas, using highly aroma-concentrated calibration samples either alone or in mixture. Chromatographic analyses were carried out in biological triplicate and technical duplicate.

The oven program started at an initial temperature of 40°C for 3 min. The temperature was then increased at a rate of 7°C min^−1^ up to 230°C. A 0.8 mm I.D. liner was used and maintained at 270°C in splitless injection mode. The carrier gas was helium at 1.0 ml·min^−1^ (99.996%). Ionization was performed by electronic impact (EI), with the electron multiplier set to 1,600 eV. The temperatures used were 200°C for the trap, 60°C for the manifold, and 280°C for the transfer line. The compounds were quantified in selected ion storage (SIS) mode, by selecting the appropriate ion masses for each compound: furfural (95 + 96), guaiacol (109 + 124), whisky lactone (99), eugenol (164), 3,4-dimethylphenol (107 + 122), vanillin (151 + 152). The wood component of white wines was calculated by summing the concentrations of furfural, guaiacol, eugenol, vanillin, cis, and trans whisky lactone, and were expressed in μg·L^−1^.

### Statistical Treatment

The chemical analyses relating to the wine compounds grouped as fermentative, grape, and wood components were statistically treated by a one-way ANOVA and a Principal Component Analysis (PCA) to statistically validate the chemical transfers occurring in the biofilm fermentations. Relationships between concentrations and PARAFAC components were explored by Partial Least Squares Discriminant analyses and their VIP score values (33). All the statistical analyses were carried out using OriginPro 8.0 (Origin Lab).

## Results and Discussion

### Classical Monitoring of Wine Fermentations

To improve the malolactic fermentation of wines, the biofilm development of an industrial strain was tested. The biofilm development of the Biolaffort strain was studied at 3 and 6 days on stainless steel and wood surfaces. The enumeration performed evidenced an average of 4.75 × 10^6^ and 8.08 × 10^6^ CFU/cm^2^ at 3 and 6 days, respectively, on stainless steel. An increase in the quantity of biofilm cells was observed on a wood support compared to a stainless steel support with 3.56 × 10^7^ and 1.45 × 10^8^ CFU/cm^2^ (data not shown). This quantification and the support effect were consistent with the data of Bastard et al. (6), which also demonstrated the presence of microcolonies after 3 days of culture, and bacteria developed in biofilm (cells trapped in a polymeric matrix) at 6 days. For this work, 6 days biofilms were chosen since they represents the best compromise between a mature biofilm phenotype and a reasonable biofilm production time.

**Figure 1:**
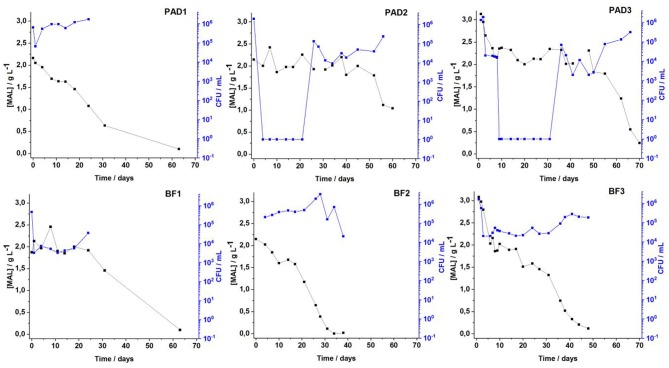
Evolution of malolactic fermentation of malic acid concentration (black legend) and the increase of the *O. oeni* cell count (blue legend) over time comparing planktonic adapted PAD (upper part of the figure) vs. biofilm BF (lower part of the figure) lifestyles adopted by *O. oeni* for the three enological conditions in wine.

To evaluate the performance of cells in biofilms in the malolactic fermentation process, classical enological analyses were checked twice a week in all the BF/PAD fermentations. Alcoholic fermentation was rapidly achieved in <1 week for the three conditions regardless of the moment of adding *O. oeni*, showing that even in the coinoculation Condition 3, the metabolic activity of *O. oeni* does not modify the activity of fermentative *Saccharomyces cerevisiae* yeasts. Very few differences were observed for the coinoculation modality, although in the literature some authors found differences in fermentation kinetics due to bacterial metabolism and interaction, both of which can affect alcoholic fermentation (34–36). Malolactic fermentation (MLF) monitoring is illustrated in Figure 1. Despite the adaptation process, very high mortality of *O. oeni* floating cells was observed, suggesting harsh conditions in the wine. In the PAD 2 and PAD 3 conditions, the wine medium had to be reinoculated for MLF to be completed after 60 days. Fermentation monitoring showed decreases in malic acid concentration from 2.2 to 1 g·L^−1^. For the biofilm condition (BF in Figure 1), malolactic fermentation was completed in <30 days and *O. oeni* adopted a physiological state, enabling it to survive and develop in wine conditions. This result was previously validated in our laboratory, highlighting the appropriateness of *O. oeni* biofilm for completing MLF (6). Analytical measurements of MLF monitoring are shown in Table S.I.1 for all conditions. Table S.I.2 indicates the shorter times to complete AF and MLF for the three BF vs. PAD for the three fermentation conditions.

### Novel Realtime Optical Monitoring of Wine Fermentations

**Figure 2:**
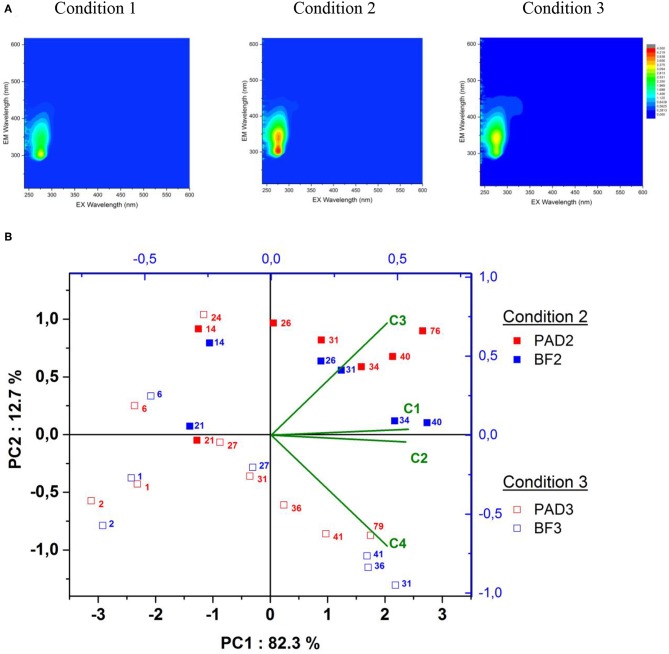
**(A)** Excitation Emission Matrices of Fluorescence analyzed on Chardonnay wines for each condition under the PAD modality after 73 days fermentation. The top left of the color scale represents the fluorescence intensity from 0 (blue) to 4.5 (red) and **(B)** The principal component analysis representing the PARAFAC components' Fmax distribution across oaked wine malolactic fermentation analyzed at different increasing fermentation times in days (numerical superscript from 1 to 79). Filled squares represent PAD (red symbols)/BF (blue symbols) for Condition 2 and empty squares for Condition 3. The loadings of the four PARAFAC components C1, C2, C3, and C4 are also indicated.

Wine malolactic fermentation was monitored by acquiring Excitation-Emission Matrices of Fluorescence (EEMF) in real-time throughout the fermentation. These 3D-map representations are illustrated in Figure 2A after 73 days of fermentation and enable describing wine fluorophore composition. This robust and reliable methodology presents the advantage of discriminating winemaking practices like sulfite addition to must (30). In this study, it was applied across wine elaboration in the different conditions, with the objectives of (i) distinguishing the enological conditions, and (ii) evaluating the impact of bacterial biofilm on wine fluorophore composition. In our study, the EEMF of white wines resulting from each condition (Figure 2A) was characterized by a broad emission band from 300 to 450 nm, with excitation wavelengths ranging from 250 to 300 nm, as described previously (30). Condition 1 was clearly differentiated from Conditions 2 and 3 by the absence of two emission contours centered at Em/Ex = 350/280 nm and Em/Ex = 450/320 nm, undoubtedly due to the absence of oak in terms of extraction and microbial interaction at the oak wood interface under enological Condition 1.

To evaluate more subtle differences occurring in Conditions 2 and 3 for the PAD/BF modalities, a wine PARAFAC statistical model was built and constrained to non-negative values and validated with four components representing 90% of the total variability of the wines analyzed (Figures S.I.1A,B). The validations of these four PARAFAC components were also split-half validated using four consecutive splits (Figures S.I.1C,D). Each wine was described by a trilinear combination of these four components, with specific scores multiplied by the maximum excitation and maximum emission loadings of each PARAFAC component, called Fmax. Each Fmax value of the four PARAFAC components for all the wines is given in Table S.I.3 and represented here in a PCA biplot for the wood containing BF/PAD conditions in Figure 2B. With this representation, a total of 95% of the overall variability is explained with the two first principal components.

Three important results were highlighted within this PCA. BF/PAD conditions present increasing values of Fmax for the four PARAFAC components while wine malolactic fermentation is occurring.

Firstly, Condition 3 after 1, 2, and 6 fermentation days: the resulting BF/PAD samples were distinguished by lower values of all PARAFAC components, undoubtedly because alcoholic fermentation was still in progress in the must and completed only after 10 days fermentation. At this stage, in Condition 3, the BF and PAD modalities were not distinguished, thereby demonstrating very close chemical composition of the wine and little impact of the presence of biofilm during fermentation.

Secondly, for both Conditions 2 and 3 and from the 26th day of fermentation until the last day of wine monitoring, BF modalities were clearly differentiated along the second component PC2 of the PCA once alcoholic fermentation was finished and malolactic fermentation was still in progress. Condition 2 was richer in PARAFAC components C1, C2, and C3 and poorer in PARAFAC component C4 compared to Condition 3. During this ongoing malolactic fermentation, no differentiation of Conditions 2 and 3 for the PAD modality was evidenced due to the high mortality of *O. oeni* and re-inoculation so that malolactic fermentation could be properly completed.

Thirdly, once malolactic fermentation was finished, Condition 2 was still discriminated from Condition 3 for both modalities (BF2/PAD2) and (BF3/PAD3) at fermentation days (40/76) and 41/79), respectively. BF fermentations were also distinguished with higher Fmax values for the four PARAFAC components. These changes in Fmax values highlight the modifications in the chemical transfers occurring through the three-dimensional structure of the biofilms compared to floating cells during MLF fermentations. Additionally, the impact of biofilm on the chemical composition of wine seemed to be greater in Condition 2 compared to Condition 3, as illustrated by the larger difference between BF and PAD wines. Therefore, the moment when biofilm malolactic fermentation occurs during wine elaboration is a crucial point in the management of the fermentative processes due to greater differentiation between wines. To go further in investigating the role of biofilms during winemaking, we decided to analyze three major components of wine, namely grape and fermentative and wood components, in the resulting wines to highlight the chemical modifications generated.

### Biofilm: A Non-selective Chemical Trapping Matrix

**Figure 3:**
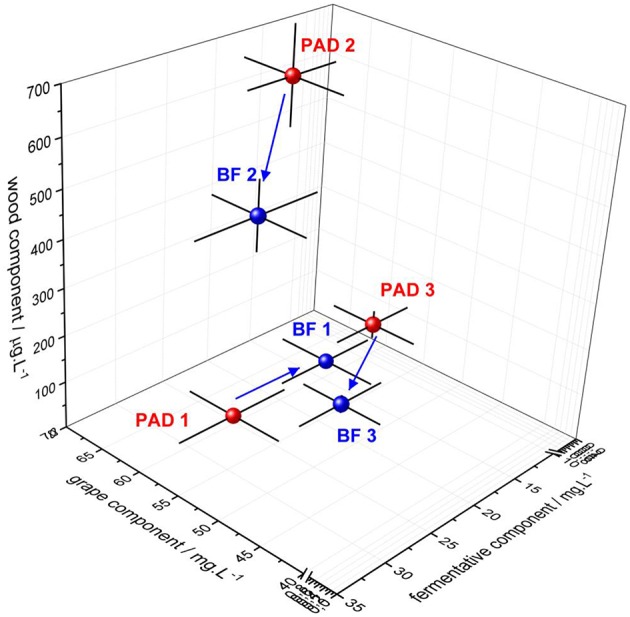
Three-dimensional representation of the concentration of wood, grape, and fermentative components for the three BF/PAD conditions. Red bubbles represent the planktonic lifestyle and blue bubbles the sessile lifestyle. For each condition, a three-dimensional error bar is indicated for each component analyzed. The blue arrow indicates the chemical transfers occurring from PAD to BF fermentation for the three conditions.

As illustrated in Figure 3 and detailed in Table S.I.4, the concentrations of wood and grape components were significantly reduced in the wines resulting from biofilm conditions in the presence of oak chips (BF2 and BF3) compared to their respective planktonic conditions (PAD2 and PAD3). These changes in the concentrations of these two components revealed retention mechanisms occurring in the biofilm that adhered to the surface of oak wood, but without any chemical specificity. Among the chemical compounds closely associated with this trapping activity of biofilm, furfural and vanillin, eugenol and guaiacol are the major wood aromas released by wood that present concentrations that fell significantly when bacterial biofilms were used under the BF modalities for Conditions 2 and 3. Concerning grape components, GRP and caftaric acid were also substantially trapped in the biofilm matrix for Conditions 2 and 3. Tyrosol was also trapped by the biofilm matrix for Condition 2 but was unaffected in Condition 3. In agreement with these results, molecular sorption in wood in the absence of bacteria was found previously for phenolic volatiles and wine hydrophobic compounds, depending on their accessibility to wood lignin binding sites (37, 38).

Whisky lactone presented distinct types of transfers under BF winemaking conditions compared to its respective PAD modality: trapping of more than half of its concentration for Condition 3, but the release of a third for Condition 3. This modulation of aromatic wood perception in wine has already been described for this compound (6), illustrating bioactivity present in the biofilm matrix. Likewise, vanillin has also been shown to react in the presence of oak wood and yeasts to form the odorless vanillic acid, and participate in the decrease observed under oaked fermentations in Conditions 2 and 3 (39, 40). In our oaked BF Conditions 2 and 3, vanillin concentrations decreased by a half and a third, respectively, highlighting that biofilm trapping activity alone is not sufficient to explain the change in concentration observed. Bacterial enzymatic activities, particularly glycosidase, have been signaled to hydrolyze wood vanillin glycosidic precursors in PAD conditions during MLF in the presence of wood (41). However, to our knowledge, this has never been evaluated in BF conditions and must be explored further in the future.

### Biofilm: A Site of Fermentative Bioactivity

As illustrated in the 3D-plot in Figure 3, the molecular composition of the resulting wines depended on bacterial lifestyle (PAD/BF) across fermentation. The fermentative component increased in the presence of oak (detailed individually for each higher alcohol in Table S.I.4). In the absence of oak (PAD1/BF1), sessile bacteria led to lower biosynthesis of higher alcohols by wine yeast, suggesting that wood/bacteria interaction may be required to increase the metabolic activity of yeasts. Although this mixed oaked fermentation has never been shown for bacteria/yeast, it had already been described in the absence of wood for *Saccharomyces cerevisiae/*non-*Saccharomyces cerevisiae* species (42). The co-inoculation of *Saccharomyces cerevisiae* and *O. oeni* under unoaked winemaking conditions has also been shown to affect the metabolic gene expression of *S. cerevisiae*, changing the aromatic profile of the resulting wines (43). Our results also highlight the increased metabolic activity of sessile bacteria in the presence of oak during fermentation, possibly attributable to enzymatic activities and changes in the transcriptome of *S. cerevisiae*, as suggested previously (43).

### Relationships Between the Optical Monitoring of Enological Conditions and Wine Chemical Composition

To define the relationships between molecular features and optical indexes associated with the biofilm trapping and metabolic activity defined previously, we analyzed the VIP scores of the partial least squares of the concentrations of the individual chemical compounds analyzed, by taking the Fmax values of PARAFAC components as the dependent variable. The results are shown in Table 3.

**Table 3:** The results of the VIP values from the partial least square applied to the concentrations in the oaked wines analyzed, resulting from Conditions 2 and 3 associated with the Fmax values of the PARAFAC components C1, C2, C3, and C4.

	**C1**	**C2**	**C3**	**C4**
Grape reaction product	**1.280**	**1.280**	**1.240**	0.654
Phenolic acids	0.821	0.662	**1.02**	**1.68**
Cinnamic acids	**1.190**	**1.110**	**1.260**	**1.410**
Tyrosol	**1.130**	**1.110**	**1.100**	0.333
Flavan-3-ols	0.201	0.077	0.582	**1.790**
Furfural	**1.150**	**1.050**	**1.240**	**1.490**
Guaiacol	**1.050**	**1.110**	**1.053**	0.434
Cis-whisky lactone	0.641	0.512	0.712	0,520
Trans-whisky lactone	0.919	**1.04**	0.798	0.542
Eugenol	**1.220**	**1.270**	**1.140**	0.368
Vanillin	**1.040**	**1.110**	0.946	0,447
Phenylethanol	0.949	**1.070**	0.826	0,444
Methanol	0.601	0.418	0.810	**1.460**
Propanol	**1.100**	0.905	**1.180**	**1.460**
2-methylpropanol	0.890	0.929	0.833	0,615
2-methylbutanol	0.759	0.852	0.698	0.933
3-methylbutanol	**1.040**	**1.060**	0.975	0.366

*Bold values are those higher than 1 and indicate a higher level of correlation between the PARAFAC components and the wine chemical concentrations*.

The level of correlation was evaluated according to the VIP score values, following a recently published procedure (33). In our experiment, chemical compounds and families like cinnamic acids and furfural were all correlated to the four PARAFAC components, meaning that these chemicals were the most statistically validated compounds associated with differentiation between PAD and BF conditions, regardless of enological Conditions 2 or 3. Among the cinnamic acids, caftaric acid, which had already been proposed as an interesting candidate related to PARAFAC components (30, 44), appeared here as a molecule easily trapped in all the BF conditions in the presence of oak. Interestingly, furfural was also found to be a good candidate, revealing the trapping activity of the biofilm and sensitive to the optical signature of evolving fermentation. To our knowledge, furfural has never been associated with the chemical significance of PARAFAC components in wines, except in the form of its derivative, 5-hydroxymethyl-furfural, previously associated with sparkling wines (45).

Separate analysis of the different enological conditions indicated that Condition 2 presented higher Fmax values for each PARAFAC component C1, C2, and C3. This was clearly associated with compounds like GRP, cinnamic acids, tyrosol, furfural, guaiacol, and eugenol. Some of these compounds have also already been proposed as chemically significant PARAFAC components (30, 44). For Condition 3, which was clearly distinguished with higher Fmax values of PARAFAC component C4, flavan-3-ols participate statistically in this differentiation of enological practices.

Although direct associations of chemicals with PARAFAC components make no sense due to the complexity of the fluorescence spectra in complex food matrices made of multiple fluorophores, our results nonetheless highlight the interest of using these PARAFAC optical indexes during wine fermentation to distinguish enological conditions applied to wine elaboration, and particularly in the case of *O. oeni* biofilm during winemaking practices.

## Conclusions

This work highlighted that regardless of the enological conditions applied to winemaking practices in the presence or absence of wood, *O. oeni* biofilm lifestyles preserve their malolactic activity in wines and confer technological properties to wine associated with the chemical transfers occurring at the wood/wine interface. In particular, grape polyphenols and wood volatile compounds are retained under all the biofilm conditions. Using a novel *in-situ* 3D-fluorescence methodology, the optical monitoring of wine fermentations was particularly reliable in distinguishing enological conditions. These fluorescence indexes made it possible to perform non-targeted differentiation between planktonic and biofilm lifestyles, suggesting that additional specific transfers and reactions across the biofilm should occur in wine, particularly in terms of grape and wood compound concentrations, and participate in qualitative changes in the resulting wines. Further studies will focus on the characterization of biofilm lifstyles in different enological conditions using different grape varieties and different lactic acid bacteria to perform malolactic fermentation to generate novel wine styles and validate their sensorial perceptions. On the other hand, a focus can be made on the nature of interactions between wine compounds and biofilm to assess possible biological activity specific to biofilm.

## Author Contributions

CC, HA, and SW conceived and designed the experiments. LP performed the experiments. CC analyzed the data and wrote the manuscript. RG and JG took part to the discussions. All the authors contributed to the final version of the article.

### Conflict of Interest Statement

The authors declare that the research was conducted in the absence of any commercial or financial relationships that could be construed as a potential conflict of interest.
