# Supplementary material for: Chemical Transfers Occurring Through Oenococcus oeni Biofilm in Different Enological Conditions
Source: Front Nutr. 2019 Jun 25;6:95. doi: 10.3389/fnut.2019.00095 (PMC6603213; doi:10.3389/fnut.2019.00095)
Supplement: Supplementary file 1 [file Data_Sheet_1.docx]

**Supplementary Information**

Chemical transfers occurring through *Oenococcus oeni* biofilm in different enological conditions

Christian Coelho^1, *^, Régis D. Gougeon^1^, Luc Perekelkine^2^, Hervé Alexandre^3^, Jean Guzzo^3^, Stéphanie Weidmann^3^

^1^UMR A 02.102 PAM Laboratoire PCAV AgroSup Dijon, Université de Bourgogne, Institut Universitaire de la Vigne et du Vin Jules Guyot, rue Claude Ladrey, BP 27877, 21078 Dijon Cedex, France

^2^SAAT Sayens, Maison régionale de l'innovation, 64a Rue Sully, 21000 Dijon

^3^UMR A 02.102 PAM Laboratoire VAlMiS AgroSup Dijon, Université de Bourgogne, Institut Universitaire de la Vigne et du Vin Jules Guyot, rue Claude Ladrey, BP 27877, 21078 Dijon Cedex, France.

Table S.I.1 : Oenological parameters followed during fermentation for the three oenological conditions. Among the parameters, TA and VA represent respectively total acidity in g/L in H_2_SO_4_ and volatile acidity in g/L in CH_3_COOH . Glu/Fru represents the ratio of glucose/fructose.

A) Condition 1 :

PAD1 :

| Time / days | 10 | 11 | 14 | 18 | 21 | 24 | 28 | 34 | 41 |
| --- | --- | --- | --- | --- | --- | --- | --- | --- | --- |
| Malic acid g/L | 2,16 | 2,05 | 1,96 | 1,69 | 1,63 | 1,63 | 1,46 | 1,08 | 0,63 |
| O.o UFC/mL | 6,44E+05 | 6,67E+04 | 5,40E+05 | 9,67E+05 | 9,67E+05 | 6,00E+05 | 1,20E+06 | 1,73E+06 | - |
| Ethanol % | 12,4 | 12,92 | 12,44 | 13 | 13 | 13,0 | 12,98 | 12,88 | 12,94 |
| TA g/L | 4,8 | 4,82 | 4,8 | 4,81 | 4,78 | 4,74 | 4,66 | 4,61 | 4,61 |
| Glu/Fru | 0,0 | 0,0 | 0,2 | 0,0 | 0,0 | 0,0 | 0,0 | 0,0 | 0,0 |
| pH | 3,23 | 3,20 | 3,21 | 3,25 | 3,24 | 3,24 | 3,26 | 3,28 | 3,32 |
| VA g/L | 0,31 | 0,43 | 0,43 | 0,46 | 0,45 | 0,44 | 0,46 | 0,46 | 0,51 |

BF1 :

| Time / days | 10 | 11 | 14 | 18 | 21 | 24 | 28 | 34 | 41 |
| --- | --- | --- | --- | --- | --- | --- | --- | --- | --- |
| Malic acid g/L | 1,87 | 2,13 | 1,97 | 2,46 | 1,90 | 1,85 | 2,00 | 1,92 | 1,46 |
| O.o UFC/mL | 4,67E+05 | 3,33E+03 | 7,33E+03 | 5,33E+03 | 3,33E+03 | 4,33E+03 | 5,67E+03 | 3,67E+04 | - |
| Ethanol % | 12,58 | 12,88 | 12,87 | 12,96 | 12,97 | 12,91 | 12,81 | 12,17 | 12,76 |
| TA g/L | 4,8 | 4,8 | 4,8 | 4,8 | 4,9 | 4,9 | 4,9 | 4,9 | 4,9 |
| Ac.malique | 2,1 | 2,1 | 2,1 | 2,1 | 2,2 | 2,2 | 2,2 | 2,0 | 2,0 |
| Glu/Fru | 0,0 | 0,0 | 0,0 | 0,0 | 0,0 | 0,0 | 0,0 | 0,0 | 0,0 |
| pH | 3,20 | 3,24 | 3,23 | 3,25 | 3,25 | 3,25 | 3,27 | 3,33 | 3,30 |
| VA g/L | 0,41 | 0,44 | 0,44 | 0,46 | 0,44 | 0,45 | 0,45 | 0,39 | 0,49 |

B) Condition 2 :

PAD2 :

| Time /days | 10 | 14 | 17 | 21 | 26 | 31 | 34 | 40 | 52 | 56 | 60 | 63 | 69 | 76 |
| --- | --- | --- | --- | --- | --- | --- | --- | --- | --- | --- | --- | --- | --- | --- |
| Malic Acid g/L | 2,15 | 1,98 | 1,98 | 2,26 | 1,93 | 1,92 | 2,01 | 1,80 | 1,79 | 1,12 | 1,04 | 1,06 | 0,32 | 0,01 |
| O.o UFC/mL | 2,00E+06 | 1,00E+00 | 1,00E+00 | 1,00E+00 | 1,33E+05 | 1,37E+04 | 9,33E+03 | 1,90E+04 | 9,33E+04 | 3,33E+05 | 6,67E+05 | 1,33E+06 | 4,67E+05 | - |
| Ethanol % | 12,9 | 12,93 | 12,9 | 12,9 | 12,92 | 12,92 | 12,78 | 12,88 | 12,96 | 12,93 | 12,83 | 12,96 | 12,7 | 12,75 |
| TA g/L | 5,2 | 4,97 | 4,99 | 4,99 | 5,06 | 5,00 | 4,97 | 4,95 | 4,88 | 4,8 | 4,75 | 4,2 | 4,51 | 4,4 |
| Ac.malique | 1,9 | 2,1 | 2,1 | 2,1 | 2,2 | 2,1 | 2,2 | 2 | 1,8 | 1,4 | 1,3 | 1,3 | 0,6 | 0,5 |
| Glu/Fru | 0,4 | 0,0 | 0,0 | 0,0 | 0,0 | 0,0 | 0,0 | 0,0 | 0,0 | 0,0 | 0,0 | 0,0 | 0,0 | 0,0 |
| pH | 3,13 | 3,23 | 3,21 | 3,22 | 3,22 | 3,24 | 3,26 | 3,25 | 3,26 | 3,28 | 3,28 | 3,26 | 3,29 | 3,32 |
| VA g/L | 0,41 | 0,44 | 0,43 | 0,45 | 0,47 | 0,44 | 0,46 | 0,47 | 0,46 | 0,49 | 0,49 | 0,46 | 0,43 | 0,51 |

BF2 :

| Time /days | 10 | 14 | 17 | 21 | 24 | 26 | 31 | 34 | 38 | 40 | 44 | 48 |
| --- | --- | --- | --- | --- | --- | --- | --- | --- | --- | --- | --- | --- |
| Malic Acid g/L | 2,15 | 2,02 | 1,84 | 1,60 | 1,67 | 1,58 | 1,17 | 0,64 | 0,39 | 0,11 | 0,00 | 0,02 |
| O.o UFC/mL |  | 2,10E+05 | 2,73E+05 | 3,83E+05 | 4,67E+05 | 4,00E+05 | 4,93E+05 | 1,93E+06 | 3,30E+06 | 1,63E+05 | 7,00E+05 | 2,07E+04 |
| Ethanol % | 12,87 | 13,07 | 12,66 | 12,89 | 12,97 | 12,86 | 12,81 | 12,99 | 12,84 | 12,87 | 12,99 | 12,72 |
| TA g/L | 5,2 | 5,1 | 5,1 | 5,0 | 4,9 | 4,9 | 4,7 | 4,6 | 4,4 | 4,3 | 4,4 | 4,4 |
| Ac.malique | 1,9 | 1,9 | 2,0 | 1,8 | 1,7 | 1,6 | 1,3 | 1,0 | 0,5 | 0,2 | 0,2 | 0,4 |
| Glu/Fru | 0,4 | 0,0 | 0,0 | 0,0 | 0,0 | 0,0 | 0,0 | 0,0 | 0,0 | 0,0 | 0,0 | 0,0 |
| pH | 3,13 | 3,17 | 3,16 | 3,17 | 3,22 | 3,22 | 3,25 | 3,24 | 3,25 | 3,29 | 3,30 | 3,32 |
| VA g/L | 0,41 | 0,40 | 0,44 | 0,43 | 0,44 | 0,43 | 0,43 | 0,47 | 0,44 | 0,45 | 0,46 | 0,50 |

C) Condition 3 :

PAD 3 :

| Time /days | 1 | 2 | 6 | 10 | 31 | 36 | 38 | 41 | 50 | 62 | 66 | 70 | 73 | 79 |
| --- | --- | --- | --- | --- | --- | --- | --- | --- | --- | --- | --- | --- | --- | --- |
| Malic Acid g/L | 3,13 | 2,95 | 2,15 | 2,37 | 2,34 | 2,32 | 2,01 | 2,02 | 1,83 | 1,24 | 0,55 | 0,25 | 0,16 | 0,09 |
| O.o UFC/mL | 1,33E+06 | 1,90E+06 | 1,67E+04 | 1,00E+00 | 1,00E+00 | 7,00E+04 | 2,03E+04 | 2,00E+03 | 2,63E+03 | 1,27E+05 | 3,67E+05 | 4,67E+05 | 8,67E+05 | 2,50E+05 |
| Ethanol % | 0,1 | 0,8 | 12,0 | 12,63 | 12,75 | 12,73 | 12,55 | 12,71 | 12,47 | 12,73 | 12,71 | 12,75 | 12,72 | 12,75 |
| TA g/L | 4,7 | 4,9 | 5,4 | 5,16 | 4,9 | 4,96 | 4,84 | 4,86 | 4,76 | 4,42 | 4,39 | 4,31 | 4,25 | 4,23 |
| Ac.malique | 3,7 | 3,5 | 2,7 | 1,9 | 2,0 | 2,1 | 2,0 | 1,9 | 1,8 | 1 | 0,5 | 0,3 | 0,2 | 0,3 |
| Glu/Fru | 205,0 | 183,5 | 5,3 | 0,6 | 0,0 | 0,0 | 0,0 | 0,0 | 0,0 | 0,0 | 0,0 | 0,0 | 0,0 | 0,0 |
| pH | 3,20 | 3,24 | 3,10 | 3,09 | 3,09 | 3,13 | 3,10 | 3,12 | 3,12 | 3,17 | 3,18 | 3,18 | 3,19 | 3,21 |
| VA g/L | 0,24 | 0,24 | 0,23 | 0,32 | 0,33 | 0,39 | 0,34 | 0,35 | 0,35 | 0,36 | 0,37 | 0,37 | 0,36 | 0,35 |

BF3 :

| Time /days | 1 | 2 | 6 | 8 | 10 | 14 | 17 | 20 | 27 | 31 | 36 | 41 | 44 | 48 |
| --- | --- | --- | --- | --- | --- | --- | --- | --- | --- | --- | --- | --- | --- | --- |
| Malic Acid g/L | 3,08 | 2,98 | 2,03 | 1,86 | 2,02 | 1,89 | 1,91 | 1,51 | 1,46 | 1,32 | 0,75 | 0,33 | 0,21 | 0,12 |
| O.o UFC/mL | 1,60E+06 | 2,00E+05 | 2,00E+04 | 5,33E+04 | 3,67E+04 | 2,77E+04 | 2,07E+04 | 2,23E+04 | 2,67E+04 | 2,87E+04 | 9,00E+04 | 2,80E+05 | 2,03E+05 | 1,83E+05 |
| Ethanol % | 0,1 | 0,5 | 10,8 | 12,0 | 12,4 | 12,7 | 12,7 | 12,5 | 11,8 | 12,7 | 12,7 | 12,7 | 12,7 | 12,7 |
| TA g/L | 4,7 | 5,0 | 5,1 | 5,1 | 4,9 | 4,9 | 4,8 | 4,8 | 4,9 | 4,7 | 4,5 | 4,3 | 4,3 | 4,2 |
| Ac.malique | 3,7 | 3,6 | 2,3 | 2,4 | 1,8 | 1,6 | 1,6 | 1,6 | 1,9 | 1,2 | 0,8 | 0,3 | 0,2 | 0,1 |
| Glu/Fru | 205,0 | 191,4 | 26,7 | 6,1 | 1,3 | 0,0 | 0,0 | 0,0 | 0,0 | 0,0 | 0,0 | 0,0 | 0,0 | 0,0 |
| pH | 3,20 | 3,21 | 3,11 | 3,11 | 3,14 | 3,11 | 3,09 | 3,14 | 3,12 | 3,22 | 3,22 | 3,20 | 3,20 | 3,21 |
| VA g/L | 0,24 | 0,25 | 0,22 | 0,19 | 0,30 | 0,36 | 0,34 | 0,34 | 0,34 | 0,45 | 0,47 | 0,38 | 0,36 | 0,38 |

Table S.I.2 : Time required to AF and MLF to achieve based on the following criteria : Glu/Fru below 2 g.L^-1^ and L-malic acid concentration below 0.2 g.L^-1^ in the three BF/PAD conditions.

| Conditions | Time to achieve AF /day | Time to achieve MLF /day | Total Fermentation time (AF+MLF) / day |
| --- | --- | --- | --- |
| PAD 1 | 10 | 63 | 73 |
| BF 1 | 10 | 43 | 53 |
| PAD 2 | 10 | 76 | 86 |
| BF 2 | 10 | 31 | 41 |
| PAD 3 | 10 | 73 | 73 |
| BF 3 | 10 | 48 | 48 |

Figure S.I.1 : Excitation (A) and Emission (B) spectra of the four PARAFAC components obtained by a split-half analysis with four independent splits and validated for excitation spectra (C) and emission spectra (D)

| A)  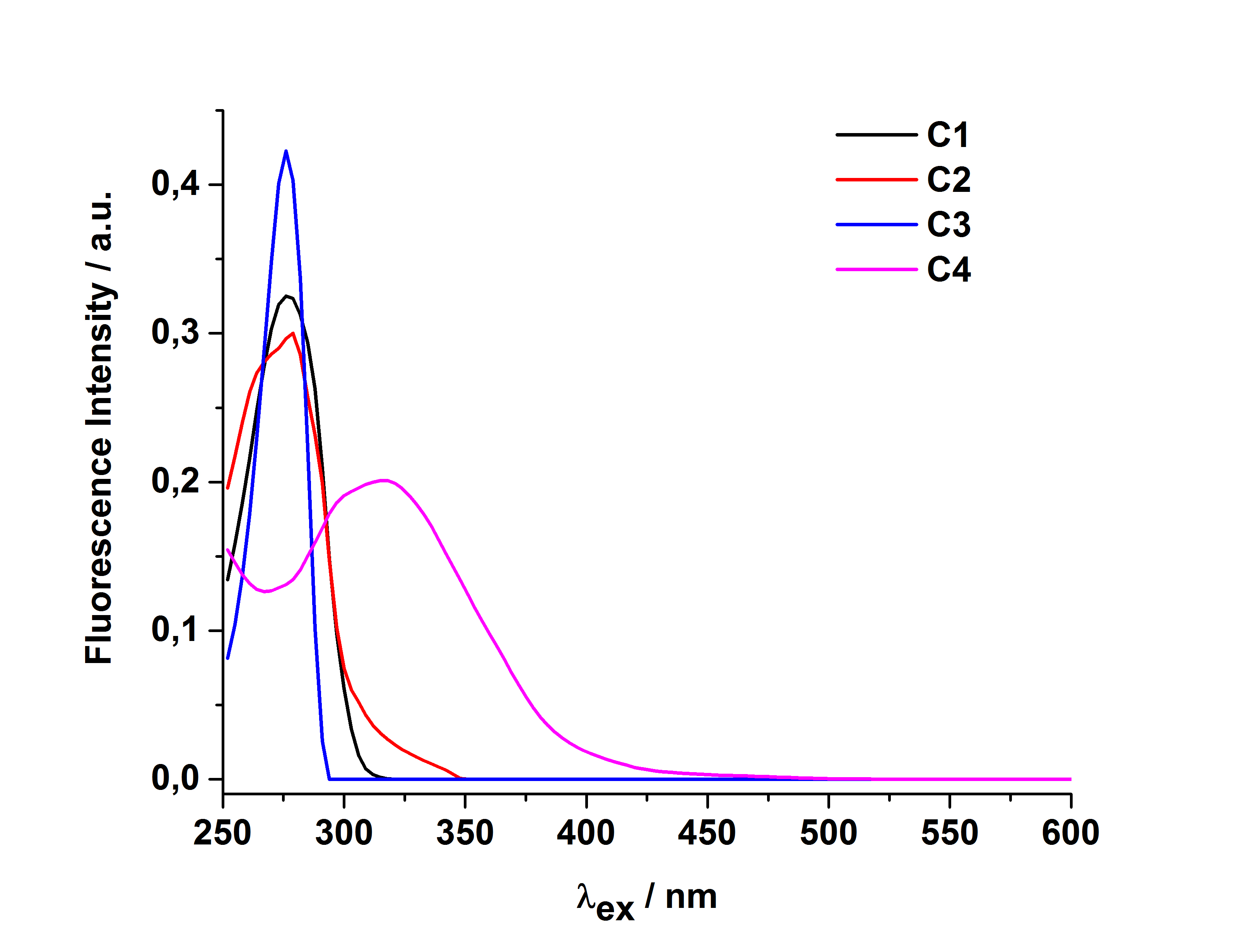 | C)  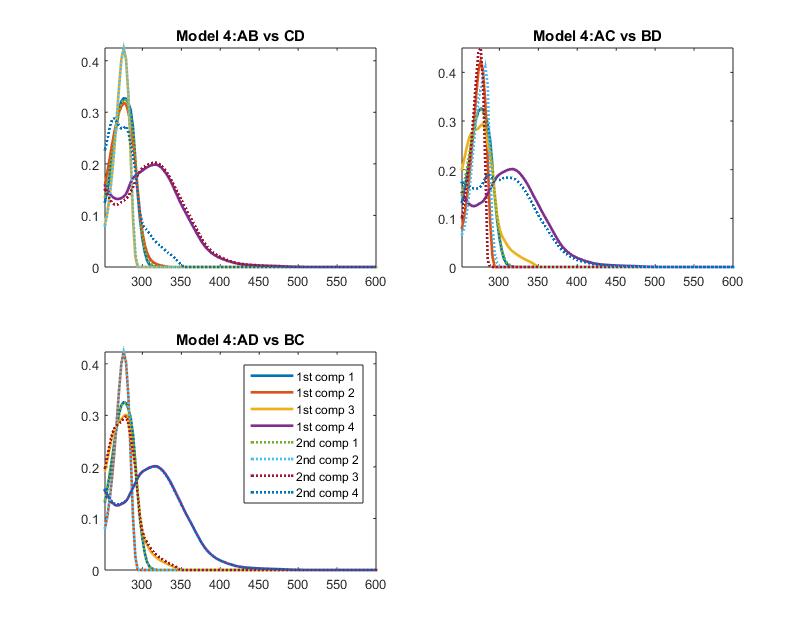 |
| --- | --- |
| B)  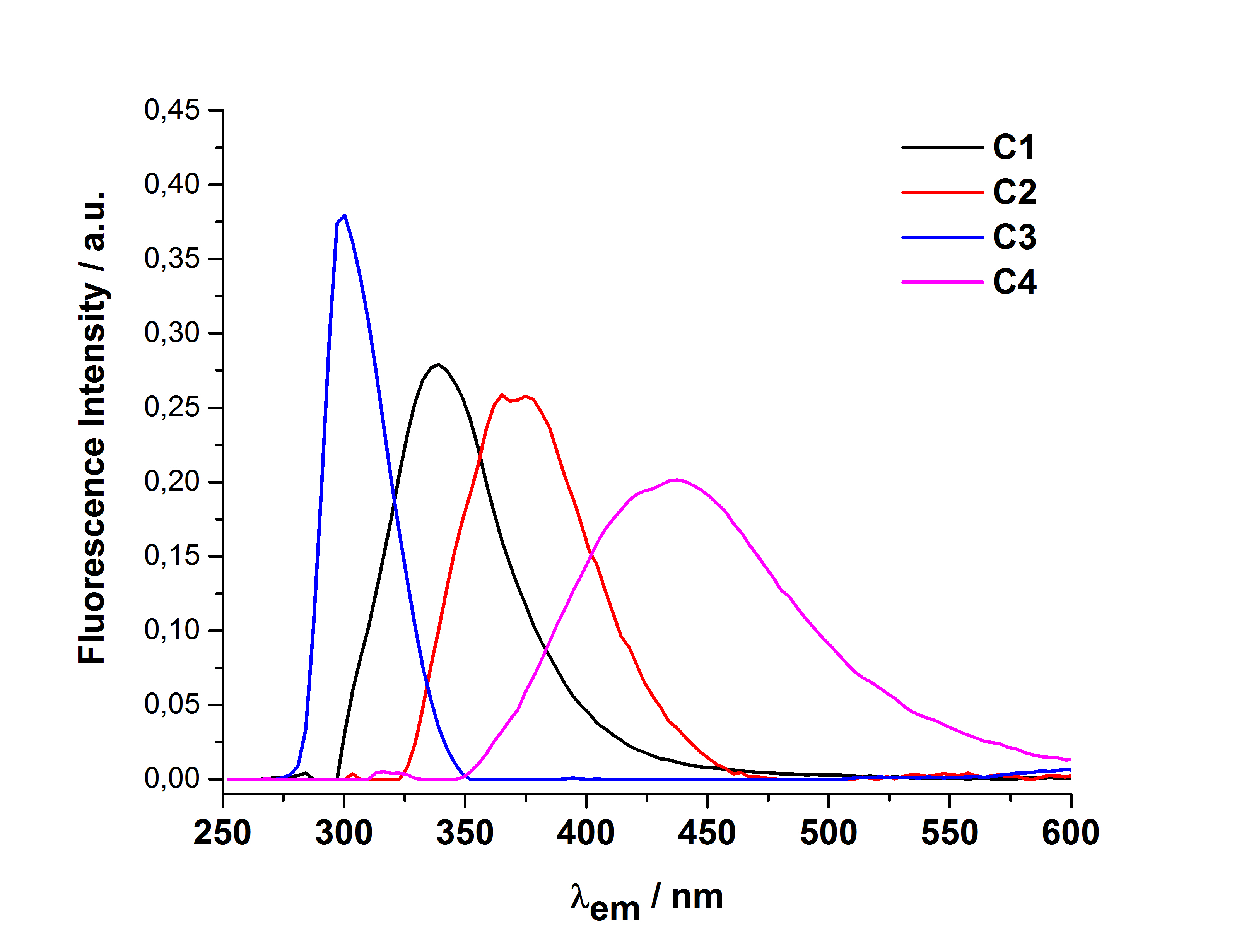 | D)  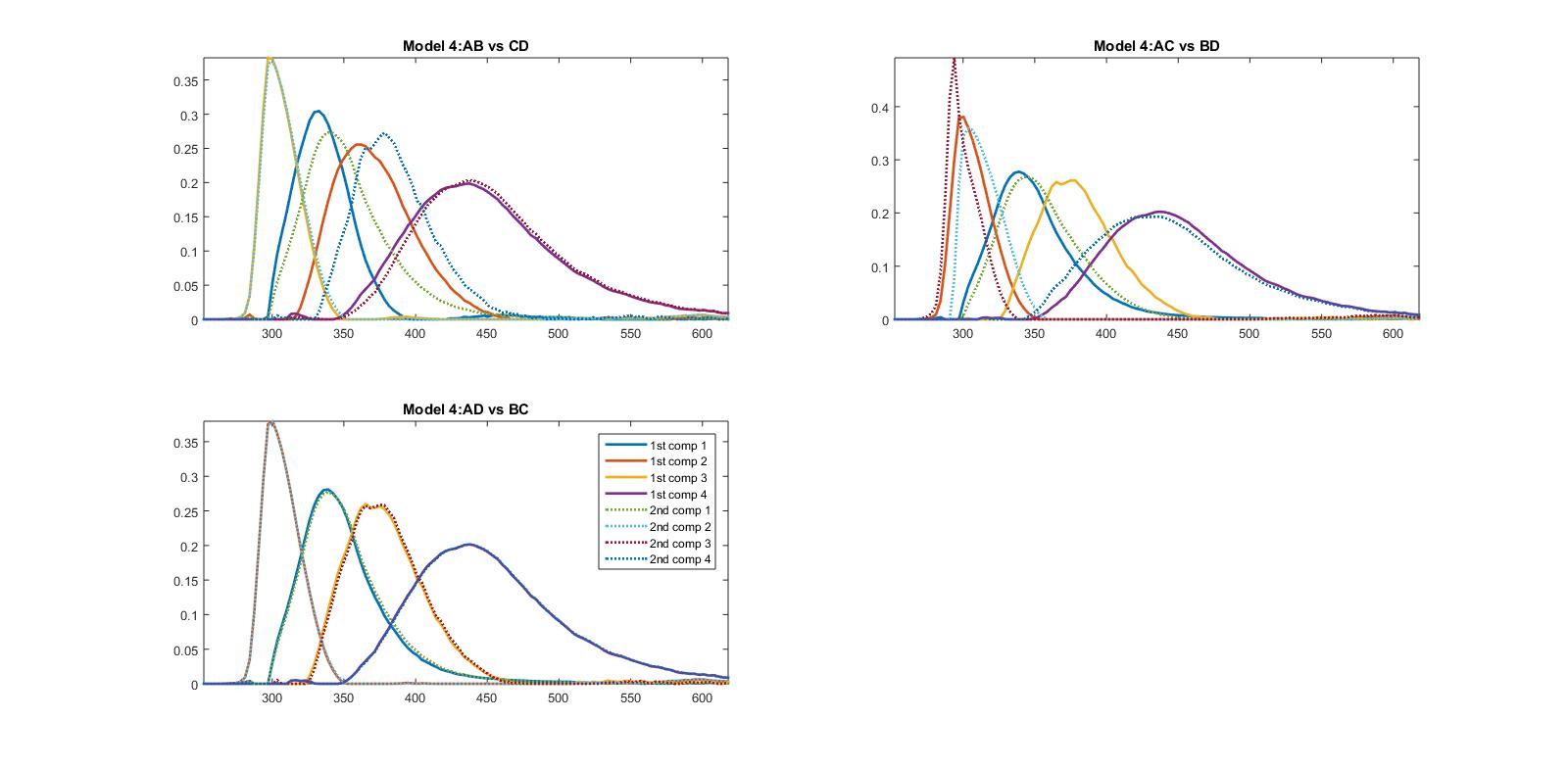 |

Table S.I.3 : Fmax values of the four PARAFAC components for Chardonnay white wines at the different fermentation days resulting from the wood containing BF/PAD Conditions 2 and 3.

| **Condition** | **Modality** | **Fermentation days** | **C1** | **C2** | **C3** | **C4** |
| --- | --- | --- | --- | --- | --- | --- |
| **Condition 2** | **BF 2** | **14** | 1,346 | 1,336 | 3,131 | 0,240 |
|  |  | **21** | 1,292 | 1,320 | 2,248 | 0,250 |
|  |  | **26** | 1,749 | 1,734 | 3,957 | 0,279 |
|  |  | **31** | 1,842 | 1,831 | 3,985 | 0,285 |
|  |  | **34** | 1,991 | 1,987 | 4,230 | 0,320 |
|  |  | **40** | 2,116 | 2,195 | 4,312 | 0,324 |
|  | **PAD2** | **14** | 1,307 | 1,297 | 3,130 | 0,232 |
|  |  | **76** | 2,370 | 2,287 | 4,237 | 0,274 |
|  |  | **21** | 1,329 | 1,347 | 2,187 | 0,255 |
|  |  | **26** | 1,600 | 1,599 | 3,711 | 0,251 |
|  |  | **31** | 1,796 | 1,771 | 3,958 | 0,268 |
|  |  | **34** | 1,911 | 1,926 | 4,148 | 0,289 |
|  |  | **40** | 2,072 | 2,133 | 4,227 | 0,286 |
| **Condition 3** | **BF 3** | **27** | 1,492 | 1,524 | 2,587 | 0,284 |
|  |  | **31** | 1,943 | 1,948 | 3,249 | 0,370 |
|  |  | **1** | 1,175 | 1,421 | 0,651 | 0,216 |
|  |  | **2** | 0,970 | 1,080 | 0,689 | 0,243 |
|  |  | **36** | 1,873 | 1,893 | 3,015 | 0,352 |
|  |  | **6** | 1,132 | 1,206 | 2,116 | 0,229 |
|  |  | **41** | 1,891 | 1,983 | 2,879 | 0,340 |
|  | **PAD 3** | **79** | 2,131 | 1,995 | 2,637 | 0,321 |
|  |  | **24** | 1,351 | 1,329 | 3,206 | 0,228 |
|  |  | **27** | 1,430 | 1,429 | 2,336 | 0,262 |
|  |  | **31** | 1,526 | 1,498 | 2,444 | 0,283 |
|  |  | **1** | 1,196 | 1,441 | 0,668 | 0,220 |
|  |  | **2** | 0,953 | 1,083 | 0,639 | 0,229 |
|  |  | **36** | 1,644 | 1,608 | 2,562 | 0,303 |
|  |  | **6** | 1,083 | 1,149 | 1,887 | 0,226 |
|  |  | **41** | 1,796 | 1,821 | 2,609 | 0,319 |

Table S.I.4 : Individual concentrations of grape polyphenols (in mg.L^-1^), wood volatile aromas (in µg.L^-1^) and higher alcohols (in mg.L^-1^) present in Chardonnay white wines resulting from the three BF/PAD conditions. Standard deviations were added for each analyte concentrations.

|  | **PAD1** | **BF1** | **PAD2** | **BF2** | **PAD3** | **BF3** |
| --- | --- | --- | --- | --- | --- | --- |
| **Grape polyphenols :** | | |  | |  | |
| Gallic acid | 0,08 ± 0.02 | 0,07 ± 0.02 | 0,39 ± 0.05 | 0,26 ± 0.04 | 1,31 ± 0.06 | 0,93 ± 0.08 |
| Protocatechuic acid | 0,36 ± 0.06 | 0,33 ± 0.08 | 0,33 ± 0.03 | 0,37 ± 0.05 | 0,42 ± 0.06 | 0,35 ± 0.05 |
| Hydroxytyrosol | 0,69 ± 0.08 | 0,58 ± 0.06 | 0,58 ± 0.03 | 0,57 ± 0.04 | 0,59 ± 0.05 | 0,56 ± 0.08 |
| Hydroxybenzoic acid | 1,27 ± 0.04 | 1,17 ± 0.04 | 1,33 ± 0.06 | 1,19 ± 0.04 | 1,32 ± 0.04 | 1,33 ± 0.05 |
| Caffeic acid | 0,84 ± 0.02 | 0,99 ± 0.04 | 0,73 ± 0.03 | 1,00 ± 0.02 | 0,67 ± 0.03 | 0,95 ± 0.03 |
| Caftaric acid | 21,59 ± 0.03 | 21,23 ± 0.03 | 22,14 ± 0.04 | 21,09 ± 0.04 | 21,34 ± 0.05 | 19,97 ± 0.04 |
| Coutaric acid | 3,35 ± 0.02 | 3,24 ± 0.02 | 3,55 ± 0.04 | 3,56 ± 0.08 | 3,03 ± 0.03 | 2,81 ± 0.05 |
| GRP | 7,59 ± 0.08 | 7,65 ± 0.07 | 7,91 ± 0.07 | 7,21 ± 0.07 | 7,49 ± 0.09 | 7,22 ± 0.08 |
| Coumaric acid | 0,41 ± 0.06 | 0,41 ± 0.06 | 0,46 ± 0.09 | 0,43 ± 0.04 | 0,45 ± 0.03 | 0,35 ± 0.03 |
| Ferulic acid | 0,07 ± 0.03 | 0,05 ± 0.04 | 0,09 ± 0.03 | 0,09 ± 0.05 | 0,10 ± 0.07 | 0,09 ± 0.07 |
| Tyrosol | 23,46 ± 0.08 | 23,11 ± 0.07 | 25,38 ± 0.09 | 22,66 ± 0.12 | 14,64 ± 0.04 | 14,88 ± 0.06 |
| (+) Catechine | 0,30 ± 0.08 | 0,30 ± 0.09 | 0,36 ± 0.06 | 0,23 ± 0.09 | 0,42 ± 0.08 | 0,44 ± 0.08 |
| (-) Epicatechine | 0,29 ± 0.06 | 0,26 ± 0.05 | 0,20 ± 0.07 | 0,18 ± 0.06 | 0,23 ± 0.07 | 0,18 ± 0.06 |
| **Wood volatile aromas :** | | |  |  |  |  |
| Furfural | 12,23 ± 1.28 | 17,08 ± 1.34 | 520,24 ± 1.43 | 374,12 ± 1.46 | 41,17 ± 1.45 | 5,74 ± 1.45 |
| Guaiacol | 0,10 ± 0.04 | 0,04 ± 0,04 | 1,02 ± 0.06 | 0,61 ± 0.06 | 1,31 ± 0.06 | 0,96 ± 0.07 |
| Cis-whisky Lactone | 3,68 ± 1.08 | 6,60 ± 1.30 | 19,39 ± 1.69 | 29,24 ± 1.80 | 21,76 ± 1.87 | 10,53 ± 1.70 |
| Trans-whisky Lactone | 0,22 ± 0.12 | 0,50 ± 0.15 | 3,08 ± 0.44 | 1,02 ± 0.39 | 5,74 ± 0.40 | 1,83 ± 0.40 |
| Eugenol | 0,66 ± 0.23 | 0,81 ± 0.22 | 2,31 ± 0.86 | 1,40 ± 0.76 | 5,35 ± 0.88 | 3,15 ± 0.70 |
| Vanillin | 7,30 ± 1.20 | 33,72 ± 1.76 | 109,60 ± 1.43 | 49,30 ± 1.40 | 151,95 ± 1.90 | 99,34 ± 1.78 |
| **Higher alcohols :** | | |  |  |  |  |
| phenylethanol | 7,43 ± 0.26 | 3,56 ± 0.30 | 6,71 ± 0.29 | 8,12 ± 0.29 | 4,38 ± 0.32 | 7,53 ± 0.29 |
| methanol | 11,85 ± 0.33 | 6,57 ± 0.32 | 7,62 ± 0.23 | 8,96 ± 0.29 | 9,91 ± 0.25 | 18,90 ± 0.34 |
| propanol | 4,26 ± 0.40 | 3,28 ± 0.23 | 2,78 ± 0.38 | 4,15 ± 0.32 | 5,83 ± 0.29 | 6,26 ± 0.30 |
| 2-methyl-propanol | 4,90 ± 0.29 | 4,30 ± 0.28 | 4,15 ± 0.19 | 5,20 ± 0.28 | 3,98 ± 0.18 | 4,33 ± 0.30 |
| 2-methyl-butanol | 8,32 ± 0.38 | 4,09 ± 0.45 | 3,58 ± 0.43 | 6,94 ± 0.41 | 3,02 ± 0.49 | 5,15 ± 0.37 |
| 3-methyl-butanol | 32,42 ± 0.29 | 28,16 ± 0.32 | 26,62 ± 0.20 | 35,55 ± 0.43 | 23,12 ± 0.30 | 26,30 ± 0.37 |
